# Parents' Knowledge and Attitudes Regarding COVID-19: Evidence From a Tertiary Healthcare Center in Irbid, Jordan

**DOI:** 10.7759/cureus.64967

**Published:** 2024-07-20

**Authors:** Nour Negresh, Liqaa Raffee, Shereen Hamadneh, Khaled Alawneh, Ruba F Al-Sheyab, Ali F Al-Shatnawi, Raya Marji, Retaj K Alawneh, Muhanad Al-Hadidi

**Affiliations:** 1 Department of General Surgery, Division of Emergency, Al-Balqa Applied University, As-Salt, JOR; 2 Department of Accident and Emergency Medicine, Jordan University of Science and Technology, Irbid, JOR; 3 Faculty of Nursing, Al Al-Bayt University, Mafraq, JOR; 4 Department of Diagnostic Radiology and Nuclear Medicine, Jordan University of Science and Technology, Irbid, JOR; 5 Department of Internal Medicine, Division of Dermatology, Al-Balqa Applied University, As-Salt, JOR; 6 Department of Internal Medicine, Jordanian Royal Medical Services, Amman, JOR; 7 Department of Pathology and Forensic Medicine, Faculty of Medicine, Al-Balqa Applied University, As-Salt, JOR; 8 Department of Accident and Emergency, Ministry of Health of Jordan, As-Salt, JOR

**Keywords:** jordan, survey, attitude, knowledge, covid-19

## Abstract

Background

Understanding parental knowledge and attitudes is crucial for effective public health interventions. This study aimed to explore knowledge and attitudes reagrding the COVID-19 crisis among parents who visited King Abdullah University Hospital (KAUH) in Irbid governorate, Jordan, during the second wave of the pandemic.

Methods

This study employed a cross-sectional design for the parents who frequently visit KAUH in the period December 2020-January 2021. A quantitative study design was used via an online survey questionnaire via WhatsApp. The data from the responses were analyzed to understand the participants' knowledge and attitudes toward COVID-19.

Results

A total of 600 parents sample participated in this study. The findings showed that around 57% of the parents had high awareness and knowledge of the different symptoms of COVID-19. Additionally, around 60% of the parents who visited the KAUH during the second wave of this crisis in December 2020 and aged from 40 to 50 years old had more awareness of the different symptoms of this virus. Knowledge and attitudes towards SARS-CoV-2 were assessed, showing that high-education respondents (n = 425) identified sneezing (80%) and touching surfaces (90%) as common transmission routes, while low-education respondents (n = 153) emphasized sneezing (100%). Preventive actions such as wearing masks (high education: 80%, low education: 100%) and avoiding crowds (high education: 80%, low education: 83.3%) were widely recognized. Both groups believed in the benefits of vitamin C-rich foods (high education: 50%, low education: 25%), but not in alcohol, for treatment. They also rejected the idea that the virus could be transmitted from mothers to fetuses or through breastfeeding. Both education levels agreed that elderly individuals are at higher risk of severe complications, with high-education respondents (90%) and low-education respondents (100%) citing severe lung infection as a major risk.

Conclusions

Generally, the sample of different age groups was more concerned with the risks and threats that could happen because of this pandemic. New studies investigating similar issues in this crisis are necessary to expand the perceptions of managing and controlling this situation.

## Introduction

In 2019, the world encountered a pandemic that has never happened in modern human history. Coronavirus disease 2019 (COVID-19) attacks the respiratory systems and lungs of humans and causes a rapid increase of confirmed infected cases over the world [1]. Like many countries worldwide, Jordan has suffered from this health crisis and confirms many experienced cases.

The growing number of confirmed cases of COVID-19 in Jordan has triggered issues regarding the levels of knowledge and attitudes the public has connected to this pandemic. Therefore, the current research aims to identify the relationship between the knowledge and attitude regarding the COVID-19 pandemic, particularly in the Irbid governorate, which has witnessed the first pandemic because of COVID-19 in the kingdom [2]. Since the first detection of coronavirus cases over the governorate of Irbid, the authorities in Jordan have taken preventive measures to curb the spread of this virus into other cities of the kingdom, consequently a full lockdown with the isolation of this governorate about other cities over Jordan [3].

Although much research and attempts have been conducted to evaluate the behavioral responses and results of COVID-19, studies in Jordan have highlighted addressing the public or patients’ knowledge and attitudes toward the perceived risks and consequences severity of this epidemic and the effective handling among their local perspective communities [4]. The process of evaluating and examining the presented knowledge and associated behaviors of the individuals in the outbreaks is considered critical, general because of the huge amount of unreliable information that misleads the public with a misconception of the COVID-19 nature and the measures needed to control and mitigate its outcomes [5]. Moreover, social media platforms and channels have a focal role in this stream, and these sources provide much information that establishes wrong perceptions among people, such as COVID-19-related conspiracy theories [3].

Jordan has adopted proactive actions with entrepreneurial actions to reduce the outbreak and outcomes of this virus. Jordan's national experience in managing this crisis has become a role model in the region since the neighborhood states have a daily basis growing of cases of COVID-19 [6]. Jordan was a better model in terms of taking protective measures than its sister countries in the Arab levant area, such as Syria, Lebanon, Iraq, and Palestine [7-9].

The authorities of Jordan have daily provided updated information regarding the local cases and the decision made to curb and control as much as they can the number of cases to remain within an acceptable range. Accordingly, the need to measure the existing levels and practices of public knowledge and attitude is highly evidenced to represent a clear evaluation of COVID-19 outcomes and the knowledge and attitudes to the preventive measures that have been taken in the entire country [10].

The main aim of this research is to examine the knowledge and attitudes of the parents who frequently visited KAUH in Irbid City (northern governorate in Jordan) regarding the possibility of being infected with COVID-19 and how they perceive their knowledge of the risks and ways of handling this public health risk.

## Materials and methods

The study used descriptive and quantitative methods to create an online survey questionnaire for data collection to achieve the study objectives. Moreover, a cross-sectional approach was adopted to conduct the study over a specific period connected with SARS-CoV-2 mainly in a determined context of KAUH in Irbid City over the second crisis in December 2020. The reasons behind this selection are the first detected cases of this virus in the kingdom of Jordan, and this city has medical pressure like the capital, which provides a great opportunity to perform such a study. Additionally, due to its availability and ease of conduct, this method is mostly recommended by many researchers to save time and effort [11]. After obtaining the phone numbers of the patients from the hospital, the researchers send the questionnaire to the patient's WhatsApp application to invite them to participate in this study. The study was conducted from December 2020 to January 2021. The Institutional Review Board (IRB) at Jordan University of Science and Technology (JUST), Irbid, approved the study. The study was performed in accordance with the principles of the Declaration of Helsinki (1975). Informed consent was waived by the IRB committee due to the retrospective nature of the study.

Study instrument

The study developed a survey questionnaire for data collection that is easy to fill and for the understanding of the demographics. The instrument has been adapted and adopted from previous related studies, which provide proper statements that could measure focal variables, such as SARS-CoV-2 knowledge and attitudes of the current study, and contribute to examining the relationships between them [12]. Accordingly, the instrument also has been validated and checked for its dependability to investigate the study concepts and what the researcher intends to measure. Thus, the content validity of the items was conducted through a panel of experts and academicians to fit the study context, and the construct validity was also analyzed to ensure reliability, grouping the items into a single factor. An online survey was created and used by social media communication platforms such as WhatsApp due to the preventive measures that have been enforced by the government, which prohibit direct contact between people, and the public fear of being infected with this virus for quick responses that could be obtained by using this data collection technique.

Ethical considerations

The research got approval from the Human Research Ethics Committee at JUST (IRB #: 2022.1.12). The researchers declared the purpose of the study, which is academic, not commercial, and ensured the confidentiality of the sample responses with anonymous participation. Additionally, the study was conducted with adherence to research ethical guidelines implemented at JUST. Participation in the study was also voluntary with no bias into any consideration such as gender or age group.

Data analysis

The study used the most common statistical software in the social sciences, namely, Statistical Product and Service Solutions (SPSS, version 29; IBM SPSS Statistics for Windows, Armonk) to analyze the collected data. The main analysis conducted was descriptive to explore the characteristics of the sample and their perceptions toward the survey items such as the percentage of the responses. Study groups were either "low educated responses", which were participants who had primary or secondary education, or "high educated responses", who had a bachelor's degree, a master of science (MSc) degree, or a doctor of philosophy (PhD) degree. A chi-square test was used for group comparison, and in the case of low cell counts, a Fisher's exact test was used. A p-value of 0.05 or less was considered statistically significant.

## Results

A total of 600 parents from different governorates in Jordan participated in this study and filled out the questionnaire. The results obtained from the sample were classified into different categories based on their educational levels, age groups, cities, and nationalities. The results showed the majority of the participants were from Irbid City (n = 449, 74.9%), which indicates the nearest university hospital for those living in Irbid City, followed by the respondents from Amman (n = 65, 10.75%) and the lowest respondents were from Jerash (n = 12, 2%), as shown in Table 1, which presents the percentage of the responses based on the cities, age group, education level, and nationality. On the other hand, the same results showed the majority hold a bachelor’s university degree (n = 368, 61.4%), followed by a secondary degree (n = 115, 19.2%). This result indicates the greater education levels among the respondents. Regarding the nationality of the respondents, the majority of the respondents were Jordanian (n = 567, 94.5%), and the least nationality was Palestinian (n = 7, 1.1%). The KAUH is a public teaching hospital and provides free healthcare services for public sector workers in Jordan, particularly for the northern cities of Jordan. In terms of the age group, the majority of the sample was aged from 20 to 30 years old (n = 241, 40.1%), followed by the age group of 30-40 years old (n = 160, 26.7%).

**Table 1:** Demographic results of the sample. * There are 22 missing responses.

Governorate	N (%)	Age group	N (%)	Nationality	N (%)	Educational level	N (%) *
Irbid	449 (74.9)	< 20yrs	33 (5.5)	Jordanian	567 (94.5)	Primary	38 (6.3)
Amman	65 (10.75)	20-30yrs	241 (40.1)			Secondary	115 (19.2)
Mafraq	45 (7.5)	31-40yrs	160 (26.7)	Syrian	26 (4.4)		
Zarqa	22 (3.6)	41-50yrs	124 (20.7)			Bachelor's degree	368 (61.4)
Jersh	12 (2.0)						
Ajloun	5 (0.9)	>50yrs	42 (7.0)	Palestinian	7 (1.1)	Master	55 (9.2)
Aqaba	2 (0.4)					PhD	2 (0.4)

The study examined the knowledge and attitudes of the sample and categorized the responses according to two categories with the same statements for each category name, educational level (high education and low education), and age group related to various aspects, such as COVID-19 symptoms, transmission, prevention, and back home actions and other considered issues with examining the existing awareness among the sample to protect their families. Table 2 shows the differences between the respondents based on their educational levels they were divided into two groups: the respondents with high education levels (bachelor, master, and PhD) and the respondents with low education levels (secondary and primary).

**Table 2:** Knowledge and attitude regarding COVID-19. ^1 ^Respondents who hold bachelor's, master's, and PhD degrees. ^2 ^Respondents who hold secondary and primary education.

Statement	High Educated (N=425) N (%)	Low Educated (N=153) N (%)	p-value
Symptoms			
Fever	425 (100)	115 (75)	<0.001
Cough	383 (90)	104 (68)	<0.001
Sore throat	255 (60)	95 (62)	0.721
Diarrhea	255 (60)	95 (62)	0.721
Transmission			
Sneezing	340 (80)	153 (100)	<0.001
Handshaking	340 (80)	153 (100)	<0.001
Touching surface	383 (90)	102 (67)	<0.001
Home preventive actions			
Avoid not going outside.	383 (90)	140 (92)	0.734
Wearing a facial mask and gloves	340 (80)	153 (100)	<0.001
Keep contact distance	383 (90)	128 (83)	0.046
Avoid crowded public places	340 (80)	128 (83)	0.385
Considerations while infected			
Contacting infected cases	298 (70)	89 (58)	0.009
Travelling to infected countries	383 (90)	115 (75)	<0.001
Having a fever	298 (70)	132 (87)	<0.001

Further, the sample was asked to explain the situations that need to be considered while infecting or diagnosing with COVID-19, which include traveling into infected places or countries, contact with confirmed cases, and having a fever (Table 2). In terms of the ways the virus could be transmitted, the respondents with low education levels (such as secondary) showed that sneezing is the most common way of transferring the virus to others. The respondents with high education levels such as university degrees believed touching contaminated surfaces is the most common means of transferring the virus. The awareness of the different educational levels of the respondents about the issues is similar, which indicates good levels of awareness among different people due to the available information and easy access to different sources, which might contribute to educating the people. The high-educated responses had higher rates of fever (100% vs. 75%), cough (90% vs. 68%), lower rates of sneezing (80% vs. 100%), and handshaking (80% vs. 100%), but a higher rate of touching surfaces behavior (90% vs. 66.6%) than lower-educated responses. Differences were statistically significant (p>0.05).

Table 3 shows the knowledge of the sample regarding the transmission and preventive measures of coronavirus. The results presented a higher percentage over the sample with a belief that some treatment could help in preventing infection with coronavirus such as eating food that is enriched with vitamin C, but taking the antibiotics would not help in this stream according to all education levels of responses. Hence, there is no difference in their perception of this item. However, they thought that old people were the group that would be infected with this virus due to their lower immunity systems and the chronic disease they have. The risky implications that could occur for the patients are varied; for instance, the educated people stated that the most implication of this virus is a severe lung infection (90%), the less educated sample agreed with this argument (100%), and no one considers that no dangerous implication will exist once an individual has become infected.

**Table 3:** Knowledge about the transmission of COVID-19. ^1 ^Respondents who hold bachelor's, master's, and PhD. ^2 ^Respondents who hold secondary and primary education.

Statement	High Educated (N=425) N (%)	Low Educated (N=153) N (%)	p-value
Elderly people are the most likely category to be infected	170 (40)	25 (16.6)	<0.001
Pregnant women are the most common category of being infected	170 (40)	13 (8.3)	<0.001
The virus might be transmitted through breastfeeding	0 (0)	25 (16.6)	<0.001
The virus might be transmitted from the mother to the fetus	0 (0)	25 (16.6)	<0.001
Risky implications are caused by coronavirus for example severe lung infection	383 (90)	153 (100)	<0.001
Taking antibiotics to reduce the chances of infection	0 (0)	0 (0)	1.0
Eating food enriched with vitamin C would reduce the chances of infection	213 (50)	38 (25)	<0.001

## Discussion

In this research, the results revealed that, during the COVID-19 pandemic, 57% of the parents who frequently visited KAUH in Irbid City had a relatively high awareness and knowledge of the different symptoms of COVID-19 and that only 20% of the parents believed the period of symptoms ranging from one week to three weeks. Additionally, 66.33% of the participants realized different ways could transmit the virus such as sneezing, touching contaminated surfaces, and others. This indicated the growing information received by the parents from different sources, which mainly focuses on educating the public about the risks of coronavirus and the ways it might spread from one person to others.

The study also found that most of the respondents 92% had recognized some essential preventive measures and methods to stay away from COVID-19. Some previous relevant research has demonstrated that the effective prevention of this virus is by staying at home and not going outside (96%). They also demonstrated that it is essential to strengthen and enhance healthcare employees and patients by supporting their capabilities to get and use reliable sources of information [13-15]. Another empirical study has proposed that increases in the number of people attending should be concentrated more on both knowledge and attitudes during the COVID-19 outbreak [16]. The present also showed that an advanced measure to protect the children during this crisis is not allowing them to go outside and contact others. Therefore, the study recommends that staying at home for children is also the first and most effective protective method for their safety since all educational institutions are closed and the government has switched to the electronic teaching approach and stopped direct personal teaching in schools and universities [17].

We found a belief among the sample of the important role of eating food enriched with vitamin C in accelerating the curing process, and alcohol also is not an effective method recommended to treat the virus based on all perspectives of the sample. The belief that COVID-19 might be transmitted from pregnant mothers to their fetuses across the placenta, or while breastfeeding the babies has no evidence over the sample, which they think is not scientifically right. About drinking herbal and taking antibiotics, the majority believed that these habits would strengthen their immunity systems regarding infection with COVID-19.

Additionally, the current research reported that, during this epidemic, around 60% of the parents who visit the KAUH and are aged 40-50 years old had more awareness of the different symptoms of COVID-19. Around 91% of the participants showed the most obvious symptom of this virus is fever followed by a cough. Other age groups also displayed greater levels of knowledge of the symptoms of this virus, which enabled them to differentiate between this virus and another disease such as influenza. Around 94% of the parents also believed that handshakes are one way of transmitting the virus between people; thus, the age groups of 20-30 years old and 40-50 years old were the most aware groups of this risk, and this means informed different ages with this issue [18].

Meanwhile, a close percentage of the parents' knowledge among different age groups regarding the ways of transmitting the virus (i.e., around 86% of participants aged above 50 years old) expressed more informative knowledge of the methods of transmission, and the most active way of this transmission was shaking hands and direct contact with people in close space. Compared with other previous epidemics such as H1N1, the COVID-19 pandemic has reported higher rates of infection and higher fatality [19]. Additionally, the detailed traits of COVID-19 are presently not clear and unknown. Thus, this reason might explain the considerations during the diagnosis process, and around 84% of the participants thought that some factors should be taken into account during this diagnosis process, such as coming from a highly infected country, contacting someone who may be infected, severe respiratory inflammation, or sudden fever. Notably, only 33% of the participants showed that only elderly people were the age group might be infected compared to the other groups because of their poor immunity system.

The results of this study are consistent with other related findings that examined the differences between genders regarding the knowledge and attitudestowards COVID-19, as the study took place in the governorate of Irbid and showed that mothers were the largest informed and knowledgeable of the COVID-19 outbreak. Additionally, they were more informed about the most common measures and concepts linked to COVID-19-like prevention, control, diagnosis, and treatment [5]. The current findings also emphasized the importance of focusing on the adoption of new advanced communication channels such as social media in light of educating the public and increasing their knowledge and attitudes toward COVID-19. This importance has come from the influential role of this method in providing and disseminating greater medical information, particularly in the time of pandemics to improve the existing knowledge and to correct the wrong practices and concepts of the people [19].

The findings were consistent with the results of a study that investigated the knowledge, practices, and attitudes of the public to COVID-19, which is considered critical to understanding the dynamic of epidemiological diseases and the effective compliance of the national measures implemented in society [20]. The study also struggled to identify the levels of attitudes and practices toward COVID-19. This study revealed good knowledge and attitude among the respondents; however, the campaigns in the health community are important to create optimistic attitudes and practices with proper intervention measurements entirely free from misconception. In the same vein, a study has evaluated the attitudes and knowledge of the physicians in the intensive care unit regarding COVID-19 and the policies and strategies adopted and responding to COVID-19 cases. The findings showed better awareness of the participants towards the subject of developing virus prevention and treatment [21].

Strengths and limitations

Our study provides a thorough documentation of parents' knowledge and attitudes regarding COVID-19 and comparisons based on education. Our study has several limitations. The current research first tried to explore the extent of parents' awareness of the threats of COVID-19 and their knowledge and attitudes during this crisis over the KAUH in the Irbid governorate in Jordan. The findings were only restricted to the identified particular sample over a certain context, which might not generalize these findings to another different setting. The time frame and the characteristics of the study were also limiting the opportunities to expand the results over other contextual studies. The study was conducted in only one public teaching hospital in one city over the kingdom, which this also considered a limitation. Other limitations include the cross-sectional design and the lack of follow-up.

## Conclusions

Generally, the sample shows greater knowledge and educated attitudes toward the SARS-CoV-2 pandemic. The major symptoms, transmission, home procedures, and protective measures were the main factors being asked the sample to examine their knowledge of this epidemic. However, the various aspects of managing this situation included personal protective equipment (PPE) usage and the right ways of prevention and measures. The recommendations for the practitioners and sector professionals would provide remarkable orientation and guidance to sufficient health information programs regarding emerging situations in society. This may help motivate the existing poor education system regarding the information access and resources available to the people. Our study has shown better knowledge and attitudes in high-educated parents compared to low-educated parents towards COVID-19.
